# Insights into the Ecotoxicology of Radicinin and (10*S*,11*S*)-(—)-*epi*-Pyriculol, Fungal Metabolites with Potential Application for Buffelgrass (*Cenchrus ciliaris*) Biocontrol

**DOI:** 10.3390/toxins15060405

**Published:** 2023-06-20

**Authors:** Antonietta Siciliano, Jesús G. Zorrilla, Lorenzo Saviano, Alessio Cimmino, Marco Guida, Marco Masi, Susan Meyer

**Affiliations:** 1 Department of Biology, University of Naples Federico II, Complesso Universitario Monte Sant’Angelo, Via Cintia 4, 80126 Naples, Italy; antonietta.siciliano@unina.it (A.S.); lorenzo.saviano@unina.it (L.S.); marco.guida@unina.it (M.G.); 2 Department of Chemical Sciences, University of Naples Federico II, Complesso Universitario Monte S. Angelo, Via Cintia, 80126 Naples, Italy; jesus.zorrilla@uca.es (J.G.Z.); alessio.cimmino@unina.it (A.C.); 3 Allelopathy Group, Department of Organic Chemistry, Facultad de Ciencias, Institute of Biomolecules (INBIO), University of Cadiz, C/Avenida República Saharaui, s/n, 11510 Puerto Real, Spain; 4 Shrub Sciences Laboratory, U.S. Forest Service Rocky Mountain Research Station, 369 North 100 West Suite 8, Cedar City, UT 84721, USA

**Keywords:** ecotoxicology, natural products, radicinin, (10*S*,11*S*)-*epi*-pyriculol, degradability, *Cochliobolus australiensis*, *Pyricularia grisea*, buffelgrass

## Abstract

Buffelgrass (*Cenchrus ciliaris* L.) is an invasive C4 perennial grass species that substantially reduces native plant diversity of the Sonoran Desert through fire promotion and resource competition. Broad-spectrum herbicides are essentially used for its control, but they have a negative environmental and ecological impact. Recently, phytotoxicity on *C. ciliaris* has been discovered for two metabolites produced in vitro by the phytopathogenic fungi *Cochliobolus australiensis* and *Pyricularia grisea.* They were identified as (10*S*,11*S*)-(—)-*epi*-pyriculol and radicinin and resulted in being potential candidates for the development of bioherbicides for buffelgrass biocontrol. They have already shown promising results, but their ecotoxicological profiles and degradability have been poorly investigated. In this study, ecotoxicological tests against representative organisms from aquatic ecosystems (*Aliivibrio fischeri* bacterium, *Raphidocelis subcapitata* alga, and *Daphnia magna* crustacean) revealed relatively low toxicity for these compounds, supporting further studies for their practical application. The stability of these metabolites in International Organization for Standardization (ISO) 8692:2012 culture medium under different temperatures and light conditions was also evaluated, revealing that 98.90% of radicinin degraded after 3 days in sunlight. Significant degradation percentages (59.51–73.82%) were also obtained at room temperature, 30 °C or under ultraviolet (254 nm) light exposure. On the other hand, (10*S*,11*S*)-*epi*-pyriculol showed more stability under all the aforementioned conditions (49.26–65.32%). The sunlight treatment was also shown to be most effective for the degradation of this metabolite. These results suggest that radicinin could provide rapid degradability when used in agrochemical formulations, whereas (10*S*,11*S*)-*epi*-pyriculol stands as a notably more stable compound.

## 1. Introduction

**Figure 1:**
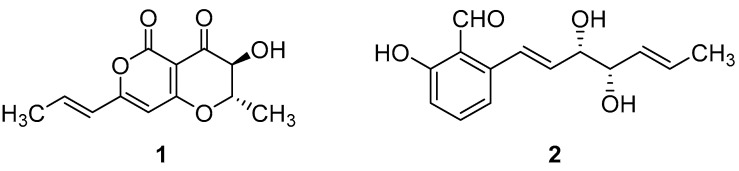
Chemical structures of radicinin (**1**) and (10*S*,11*S*)-(—)-*epi*-pyriculol (**2**).

The C4 perennial grass *Cenchrus ciliaris* L. (also known as buffelgrass) is a problematic invasive species in North America and Australia [1,2]. In particular, it has become widespread in the Sonoran Desert of Southern Arizona, where it accumulates abundant biomass that can carry fires through this ecosystem, which is not adapted to fire [3]. Furthermore, it can compete for resources, thus reducing native plant diversity [4]. This competition and the fire promotion are resulting in devastating impacts for native plants and animal life of the Sonoran Desert [5]. The control of buffelgrass is essentially on the use of broad-spectrum herbicides despite the negative environmental and ecological impact [6]. In particular, glyphosate and imazapyr are used in the Sonoran Desert, causing major damage to native species [7]. Furthermore, buffelgrass is very difficult to eradicate and requires multiple follow-up treatments to suppress populations [8]. To avoid the accumulation of toxic substances and to preserve the Sonoran Desert ecosystem, the development of a suitable bioherbicide is urgently required. Phytotoxins produced by weed pathogenic fungi could be used to design safe bioherbicides for buffelgrass management [9]. Thus, as has been the case with several phytopathogenic fungi, one of the key study points is the research on the host-selective and non-specific host toxins they produce [10]. For this reason, the fungal pathogens *Pyricularia grisea* and *Cochliobolus australiensis*, isolated from diseased leaves of buffelgrass in its North American range, have been studied to evaluate the production of potential natural phytotoxins for the biocontrol of this invasive weed. Several secondary metabolites, belonging to different classes of natural compounds, were identified, characterized, and tested for their phytotoxicity on buffelgrass. In particular, among the nine compounds obtained from in vitro cultures of *P. grisea* and the nine from *C. australiensis*, radicinin and (10*S*,11*S*)-(—)-*epi*-pyriculol (hereinafter abbreviated to “EPYR”) (Figure 1) proved to be the most promising ones [7].

Radicinin was also suggested as a key active metabolite produced by other fungi with herbicidal and fungicidal properties [11]. Interestingly, some particularities of its structure are also shown by different bioactive metabolites produced by other phytopathogenic fungi, such as the presence of a cyclic ester integrated in a conjugated system [12,13]. Indeed, it is worth highlighting that unsaturated cyclic esters are generally common moieties in diverse compounds with phytotoxic and growth-inhibitory activities [14,15]. Studies on the mode of action of radicinin were initiated in the middle of the 20th century, with reports that it had no effects on the quantity or degree of unsaturation of the fatty acids produced by *Fusarium lini*, while further studies provided the confirmation of its absolute configuration through chiroptical methods [16,17]. The use of radicinin in agriculture could also be supported by its dose-dependent inhibitory activity that is triggered by targeting protease activity against the phytopathogenic bacteria *Xylella fastidiosa* [18]. On the other hand, only a few references are available about EPYR (and its isomer pyriculol), mainly reporting its isolation from *P. grisea* or its synthesis [19,20,21,22]. However, to continue all of these studies at the whole-plant level for the development of natural bioherbicides, larger quantities of both compounds are needed. Thus, high-performance liquid chromatography (HPLC) analyses were used to select the best source and culture conditions in order to optimize their large-scale production by fungal fermentation [23,24] and prompt research on their total stereoselective synthesis [19,20,25]. Even though these two compounds (**1** and **2**) had already shown promising results, their efficiency, degradation, and ecotoxicological profiles were poorly investigated. In this study, ecotoxicological tests using representative organisms from aquatic ecosystems, i.e., *Aliivibrio fischeri* (bacterium), *Raphidocelis subcapitata* (alga), and *Daphnia magna* (crustacean), were performed for each of the two compounds. Furthermore, their degradability was evaluated to provide conclusions about their stability under specific light and temperature conditions.

## 2. Results and Discussion

To evaluate the ecotoxicological profiles of the fungal metabolites radicinin and (10*S*,11*S*)-*epi*-pyriculol, both compounds were first isolated from their respective fungal sources and identified as reported in Section 4.

### 2.1. Toxicity of Radicinin and EPYR against Representative Organisms

The ecotoxicity of radicinin and EPYR on representative organisms from aquatic ecosystems was evaluated in the range of concentrations of 0.15–41.50 mg/L, following the reported International Organization for Standardization (ISO) protocols 8692:2012 [26], 11348-3:2007 [27], and 6341:2012 [28], as detailed in Section 4.

**Figure 2:**
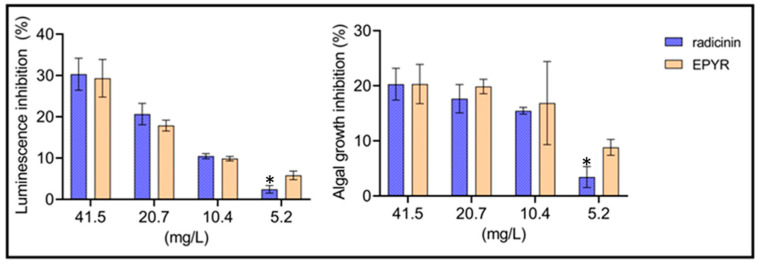
Luminescence inhibition of *Aliivibrio fischeri* and algal growth inhibition of *Raphidocelis subcapitata* after exposure to radicinin and (10*S*,11*S*)-*epi*-pyriculol (EPYR). Data represent mean and standard deviation. The level of statistical significance is denoted by asterisks: * (*p* ≤ 0.05).

*R. subcapitata*, a freshwater alga, and *A. fischeri*, a marine bacterium, did not exhibit any significant sensitivity to either compound, with toxicity levels ranging from 3 to 30% and from 5 to 29% for bacterial luminescence and from 3 to 20% and from 9 to 20% for algal growth inhibition, respectively, in a dose-dependent manner (Figure 2).

In the case of *D. magna*, another standard freshwater test organism, higher toxicity levels in response to both compounds were obtained, with median effective concentration (EC_50_) values of 19.14 mg/L for radicinin and 24.82 mg/L for EPYR, indicating relatively low toxicity levels after 24 h of exposure (Table 1). According to the United Nations Globally Harmonized System of and Labelling Chemicals [29], the results on *D. magna* suggested that radicinin and EPYR could be considered hazardous to the aquatic environment, as their EC_50_ values are below 100 mg/L (ACUTE 3 category). Nevertheless, the tested concentrations ranged from 0.15 to 41.50 mg/L; therefore, they were relatively high compared to expected environmental concentrations. Hence, these compounds may have a low environmental risk when used as pesticides. It should be noted that the tested concentrations may not fully represent the environmental concentration range, and lower concentrations may still have sublethal effects or effects on non-target species [30]. This is based on previous studies that have shown sublethal effects of similar compounds on non-target species at lower concentrations [30]. These findings highlight the importance of considering potential effects at lower concentrations and conducting further research to fully understand the ecological impacts of these compounds.

When comparing the toxic effects of radicinin and EPYR on the different aquatic organisms considered, it is interesting to note that *R. subcapitata* and *A. fischeri* did not show any sensitivity to the compounds, while *D. magna* did. This could be due to differences in the physiology and sensitivity of these organisms, as well as their different exposure routes to the compounds.

While the ecotoxicity results for radicinin and EPYR on organisms from aquatic ecosystems indicated relatively low toxicity levels, their phytotoxicity may still have significant effects on terrestrial ecosystems. In particular, in the study reported by Masi (2019) [7], it was proved that radicinin and EPYR exert significant phytotoxicity against *C. ciliaris* at a concentration of 2.5 × 10^−3^ M, while they exhibit reduced effects against a panel of native species of the Sonoran Desert. At a lower concentration of 10^−3^ M, radicinin did not show phytotoxic effects on the same native species, but it still maintained its toxicity against *C. ciliaris.* However, considering the potential use of the two compounds under study as potential herbicides, it is important to remark their low toxicity against *D. magna* at these concentrations (2.5 × 10^−3^ M and 10^−3^ M). Specifically, the toxicity of radicinin and EPYR was below their EC_5_ values at 10^−3^ M (Table 1).

The fact that radicinin has shown potential cytotoxicity against human tumor cell lines [31] raises concerns about its potential impact on human health if it enters the food chain. This highlights the importance of considering the potential effects of these compounds on a wider range of ecosystems, including terrestrial ecosystems and their native species. In this regard, it could be remarked that teratogenic, sublethal, or lethal effects on zebrafish (*Brachydanio rerio*) embryos were not found in a previous toxicological study with radicinin [7].

### 2.2. Stability Assays of EPYR and Radicinin

While further studies are needed to better understand how the study compounds interact with both target and non-target organisms, the role of the half-life on the test medium in modulating the observed trends should not be ruled out. The partial or total degradation of the natural compound may reduce its effective toxicity over time and thus decrease its risk from an environmental point of view. To examine this possibility, this study also included stability tests of both metabolites, as detailed below.

The stability of radicinin and EPYR was evaluated by quantifying their respective degradability percentage in defined media under specific temperature and light conditions: room temperature, in laboratory oven at 30 °C, under ultraviolet (254 nm) light (UV) and under sunlight. All treatments were applied for 72 h (corresponding to the longest time used for the algal inhibition test), with the UV-light test being an exception (4 h). After these periods, samples were extracted with ethyl acetate, and their respective organic extracts were subjected to qualitative and quantitative analyses to determine the amount of radicinin or EPYR and the presence of degradation products.

**Figure 3:**
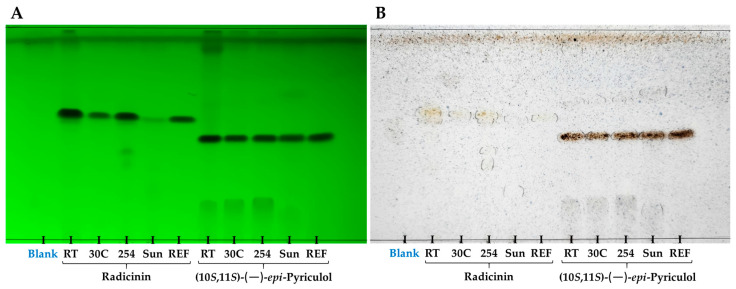
Thin-layer chromatography analysis of the organic extract of samples initially containing radicinin or (10*S*,11*S*)-(—)-*epi*-pyriculol, after 72 h at room temperature (RT), 30 °C (30C) or under sunlight (Sun), or after 4 h under 254 nm light (254). Samples of standards of both compounds (REF) and a negative control containing the ethyl acetate extract of ISO culture medium (Blank) were included. Plate eluted in CHCl_3_/*i*-propanol (9/1, *v*/*v*) and visualized (**A**) under 254 nm light, or (**B**) by spraying with 10% H_2_SO_4_ in methanol (*v*/*v*) and a 5% solution of phosphomolybdic acid in ethanol (*v*/*v*), followed by heating at 110 °C for 10 min.

The qualitative analysis was performed by thin-layer chromatography (TLC) analysis of the organic extracts, in comparison with standard samples of radicinin and EPYR. The TLC plate (Figure 3), eluted in CHCl_3_/*i*-propanol (9/1, *v*/*v*), was first visualized under UV light (Figure 3A), revealing the partial degradation of radicinin and EPYR under certain test conditions. In the case of radicinin, marked degradation was observed under sunlight, as evidenced by the less intense band in comparison with the other treatments. Moreover, at room temperature and under UV light exposure, spots corresponding to more polar degradation products were observed, with the UV-light-treated sample showing two additional products with notably lower retardation factors (*R_F_*). When the TLC plate was visualized after spraying with H_2_SO_4_ and phosphomolybdic acid and heating (Figure 3B), only slight intense sports were visualized for radicinin, indicating that this method of detection did not yield relevant information.

The TLC analysis of the degradation of EPYR showed a degradation product with a lower *R_F_* value for all the test conditions and both detection methods (Figure 3). The sunlight-exposure sample, when visualized under UV light (Figure 3A), showed an additional degradation product seen as a slightly intense spot with a higher *R_F_* than EPYR.

The quantitative evaluation of the degradation products was performed by HPLC analysis of the same organic extracts. The amount of radicinin or EPYR was quantified according to reported protocols for its quantification in complex mixtures [23,24]. The retention times were highly reproducible, showing variations of less than 0.500 min. The results are summarized in Table 2.

In agreement with the qualitative analysis, the quantitative analysis showed that both compounds degrade in the ISO medium at different rates according to the conditions applied. At room temperature, the degradation percentage is approximately half of the initial test amount, 59.51% (radicinin) and 49.26% (EPYR). These percentages increased by approximately 10% when treated at 30 °C or under 254 nm light, whereas the highest percentages were always obtained for the samples treated under sunlight. This condition was especially effective (98.90%) for the degradation of radicinin. These results agree with those already reported for the degradation of fungal toxins in carrots. It was found that the amounts of radicinin produced by *Alternaria radicina* decreased through time in stored dried carrots. This is relevant given the inhibitory activity of radicinin on the root growth of germinating carrot seeds when tested at 10−20 μg/mL [32,33]. Later, specific studies in vascular storage parenchyma cells of carrot roots indicated that, although some specific effects were tentatively noted, radicinin did not generate visible changes or changes in the structure of endoplasmic reticulum and dictyosomes when tested at 25 µg/mL [34].

**Figure 4:**
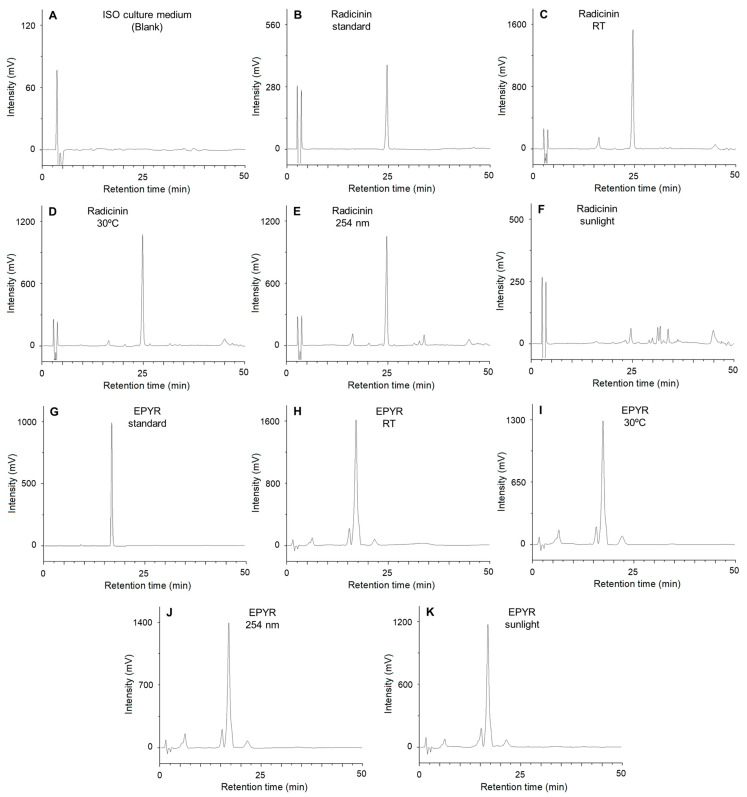
Chromatograms of (**A**) ISO culture medium (blank) extract; (**B**) radicinin standard at 0.5 mg/mL; (**C**) radicinin sample after 72 h at room temperature; (**D**) radicinin sample after 72 h at 30 °C; (**E**) radicinin sample after 4 h under 254 nm light; (**F**) radicinin sample after 72 h under sunlight; (**G**) (10*S*,11*S*)-(—)-*epi*-pyriculol standard at 1 mg/mL; (**H**) (10*S*,11*S*)-(—)-*epi*-pyriculol sample after 72 h at room temperature; (**I**) (10*S*,11*S*)-(—)-*epi*-pyriculol sample after 72 h at 30 °C; (**J**) (10*S*,11*S*)-(—)-*epi*-pyriculol sample after 4 h under 254 nm light; (**K**) (10*S*,11*S*)-(—)-*epi*-pyriculol sample after 72 h under sunlight.

The chromatograms of the quantitative analyses herein reported are depicted in Figure 4. In the case of radicinin (Figure 4B–F), a degradation product with lower retention time (16.090–16.200 min) than radicinin could be observed in the chromatograms of all the degradation tests, with the exception of the sunlight treatment (Figure 4F). On the other hand, the sunlight exposure generated the degradation of almost all the radicinin into different products with higher retention times (with peaks appearing at 31.170, 31.760, 33.720, and 44.877 min), all in similar amounts. Previous studies on the biosynthesis and synthesis of derivatives of radicinin showed that this compound possesses different reactivity sites, which would explain its ease of degradability [35]. Additionally, a study with the phytopathogenic fungus *Bipolaris coicis* proved that radicinin can epimerize at C-3 [36], from which it could be suggested that certain degradation products are derived from modifications in this position.

The degradation of EPYR (Figure 4H–K) was observed to always produce three common degradation products, two of them at lower retention times than EPYR (7.070 and 16.485 min), and one with a higher retention time (21.774 min). The aldehyde group of EPYR could be one of the most reactive moieties in its structure, which is reactive under reductive conditions that lead to the corresponding primary alcohol, as found for the structurally related compounds pyriculol and pyriculariol [37]. In fact, the corresponding alcohols and aldehydes are suggested to undergo interconversion via oxidation and reduction reactions, and their biosynthesis is a matter of study in recent reports [37,38]. Given that EPYR also contains a lateral chain with two double bonds, it could be suggested that, especially under sunlight treatment in aqueous solution, isomerization reactions could lead to degradation products with the opposite geometry of some of these double bonds. These could be produced by mechanisms such as a first hydration and later elimination, or a triplet energy transfer and later sigma-bond rotation [39].

## 3. Conclusions

According to previous studies, radicinin and EPYR are natural products with potential applicability as herbicides for the biocontrol of buffelgrass due to their phytotoxic activity. Since there are very few previous publications on ecotoxicological evaluations, more in-depth studies were necessary to provide conclusions in this regard and thereby ensure that their applicability would be in line with environmental requirements. The present study suggests that radicinin and EPYR may not pose a significant risk to aquatic ecosystems at environmentally relevant concentrations, considering the toxicological test against the representative organisms *Aliivibrio fischeri*, *Raphidocelis subcapitata,* and *Daphnia magna*. The stability study showed that both compounds in aqueous media degrade at room temperature, with sunlight exposure enhancing their degradation rates, especially for radicinin, which was almost completely degraded. Higher temperatures and UV-light exposure could also increase degradability, although to a lesser degree than sunlight exposure. Rapid degradability under ambient environmental conditions could reduce the potential environmental impacts of these compounds but will also perhaps limit their utility as bioherbicides unless methods to increase their stability can be implemented.

Further research is needed to determine the potential long-term effects of these compounds and the risks associated with their production, transport, and disposal. Comparisons with other commonly used pesticides or herbicides and natural compounds with similar properties would also provide a better understanding of their potential impact on the environment and whether they could be ideal candidates with less capacity for harm. It is crucial to consider the potential impact of these compounds on human health, as well, especially considering the previous reports about the cytotoxicity of radicinin against human tumor cell lines. Proper regulations and guidelines must be in place to ensure their safe use and disposal, and more research is needed to determine the extent of this risk.

## 4. Materials and Methods

### 4.1. Obtaining and Identification of Radicinin and EPYR

Radicinin and EPYR were isolated by chromatographic means from a *C. australiensis* or *P. grisea* strain, respectively, grown on potato dextrose broth culture, following reported procedures [23,24]. Their identification was performed by spectroscopic and spectrometric studies. In particular, a liquid chromatography/time-of-flight mass (LC/MS TOF) Agilent 6230B (Agilent Technologies, Milan, Italy) and 400 MHz NMR Bruker (Karlsruhe, Germany) spectrometers were used to record ^1^H NMR and mass electrospray ionization mass spectra (ESIMS). Their spectroscopic data were in agreement with those available in the literature for radicinin and (10*S*,11*S*)-(—)-*epi*-pyriculol [24,40]. Sigma-Aldrich Co. (St. Louis, MO, USA) supplied all solvents.

### 4.2. Toxicity Assays

The ISO 8692:2012 [26] medium was employed for the preparation of test solutions and for the control medium. This was an artificial freshwater solution containing the nutrients CaCl_2_⋅2(H_2_O), MgSO_4_⋅7(H_2_O), NaHCO_3_, KCl, Na_2_EDTA⋅2(H_2_O), NaNO_3_, NH_4_Cl, MgCl_2_⋅6(H_2_O), MnCl_2_⋅4(H_2_O), K_2_HPO_4_, FeCl_3_⋅6(H_2_O), H_3_BO_3_, ZnCl_2_, CoCl_2_⋅6(H_2_O), Na_2_MoO_4_⋅2(H_2_O), CuCl_2_⋅2(H_2_O), and NaCl [26]. All chemicals were analytical grade and supplied by Sigma-Aldrich. Ultrapure water was used to prepare dilution water and treatment.

Toxicity tests with *R. subcapitata* followed the ISO 8692:2012 protocol [26], adapted to a microplate format. The initial cell densities of *R. subcapitata* were correlated with optical density (OD) at 670 nm, according to the specification of the algae supplier, using a DR5000 spectrophotometer (Hach Lange, Weinheim, Germany). Percentages of growth inhibition were calculated according to time of exposure and concentrations compounds with respect to the controls. This assay was carried out in triplicate.

The acute ecotoxicity test with the bioluminescent bacterium *A. fischeri* was performed on a Microtox^®^ 500 analyzer (New Castle, DE, USA) according to ISO 11348-3 [27]. The bacteria used belong to the NRRL line B-11177 supplied by Microbiotests, Gent, Belgium. The diluent used was 2% saline water solution (NaCl) to osmotically adjust the salinity of the sample. The toxicity of the samples was evaluated by the bioluminescence inhibition percentage. In particular, the % inhibition was evaluated after 5, 15, and 30 min of exposure of the bacteria to serial dilutions of the compounds.

Methods for acute ecotoxicity tests with *D. magna* conformed to guidelines set by the ISO 6341 [28]. For these tests, five neonates of *D. magna* (<24 h old) were carefully pipetted into glass beakers with 10 mL of ISO medium. During acute ecotoxicity tests, the organisms were not fed. For each treatment, 20 neonates (4 beakers) were exposed to the control and to each compound. The complete immobilization of the organisms was considered to be indicative of the toxic effect and was recorded using a stereomicroscope (LEICA EZ4-HD).

### 4.3. Degradation Assays

For the room-temperature samples, 2.00 mg of radicinin and EPYR was dissolved in dimethyl sulfoxide (20 μL) in glass vials, and 20 mL of ISO culture medium was added. Samples were kept in the laboratory at room temperature, in darkness, for 72 h.

The following assays were performed with 1.00 mg of compound, 10 μL of dimethyl sulfoxide, and 10 mL of ISO culture medium. For the 30 °C assays, samples were placed into an oven at this temperature in darkness for 72 h. In agreement with the reported protocols [41], the photodegradation assays were performed under a 254 nm light exposure for 4 h and by sunlight irradiation to solar light for 72 h (Naples, Italy, February 2023).

After the corresponding time periods, the organic extract of each sample was obtained by extraction with ethyl acetate (10 mL × 3), dried over anhydrous Na_2_SO_4_, filtrated, and evaporated under reduced pressure. A sample containing 10 μL of dimethyl sulfoxide and 10 mL of ISO culture medium was similarly extracted to obtain the blank sample for the following analyses.

### 4.4. Thin-Layer Chromatography and HPLC Analyses

The TLC analysis was performed on 10 cm high silica-gel plates (Kiesel-gel 60 F254, 0.25 mm, Merck, Darmstadt, Germany). Spots were visualized by exposure to UV radiation (254 nm) and by spraying with 10% H_2_SO_4_ in methanol (MeOH) (*v*/*v*) and a 5% solution of phosphomolybdic acid in ethanol (*v*/*v*), followed by heating at 110 °C for 10 min.

The HPLC analyses were performed using a Phenomenex C18 reversed-phase column Luna (150 × 4.6 mm i.d.; 5 µm, Torrance, CA, USA) on a Hitachi HPLC system (Tokyo, Japan) paired with a 5160 pump and a 5410 spectrophotometric detector. The flow rate was 0.5 mL/min, and all samples (previously filtered through 0.45 µm filter) were analyzed in triplicates after injection through a 20 µL loop.

The analysis of radicinin was performed using an acetonitrile/H_2_O gradient, starting from 10% acetonitrile and increasing linearly to 15% in 6 min, 20% in 16 min, 25% in 22 min, 40% in 40 min, and 90% in 45 min, and then it was finally rebalanced to the initial rate for 5 min [23]. The detection was performed at 226 nm, corresponding to the maximum UV absorption reported for radicinin [42]. All radicinin samples were dissolved in methanol and analyzed at 0.5 mg/mL.

The analysis of EPYR was performed using a methanol/H_2_O (0.1% formic acid) gradient, starting from 50% methanol, increasing linearly to 70% in 30 min, and finally rebalanced to the initial rate for 10 min [24]. The detection was performed at 231 nm, corresponding to the maximum UV absorption reported for (10*S*,11*S*)-(—)-*epi*-pyriculol [21]. All EPYR samples were dissolved in methanol and analyzed at 1 mg/mL.

### 4.5. Statistical Analysis

Following the screening assays, testing concentrations were established to determine the median, 20%, 10%, and 5% effect concentrations (EC_50_, EC_20_, EC_10_, and EC_5_). These concentrations were calculated using both linear and nonlinear regression methods. The selection of the most appropriate model for compounds was based on the mean-corrected coefficient of determination (R2) and graphical evaluation of the model fit. To assess differences between treatments, a test-*t* was performed.

## Figures and Tables

**Table 1:** EC_5_, EC_10_, EC_20_, and EC_50_ values for radicinin and EPYR on *Daphnia magna*; values are in mg/L; EC = effective concentration; average EC values are provided ±95% confidence limit values in brackets (*n* = 3). EC values determined by nonlinear regression: radicinin r^2^ = 0.84; EPYR r^2^ = 0.92.

Compounds	EC_5_	EC_10_	EC_20_	EC_50_
Radicinin	0.62 (0.03–4.45)	0.86 (0.04–5.52)	1.68 (0.08–8.52)	19.14 (12.49–27.71)
(10*S*,11*S*)-(—)-*epi*-Pyriculol (EPYR)	0.49 (0.04–2.96)	0.72 (0.06–3.88)	1.57 (0.13–3.88)	24.82 (19.87–31.64)

**Table 2:** Retention times of radicinin and (10*S*,11*S*)-(—)-*epi*-pyriculol (EPYR) in the HPLC analyses, amount of both compounds detected in each analysis, and percentage of compound degraded.

Compound and Degradation Method	Concentration for Analysis (mg/mL)	Retention Time (min)	Amount of Compound Detected (µg) in 20 µL	% of Compound Degraded after 72 h
Radicinin (standard)	0.5	24.583	-	-
Radicinin (room temperature)	0.5	24.560	4.049 ± 0.011	59.51
Radicinin (30 °C)	0.5	24.603	2.629 ± 0.004	73.71
Radicinin (254 nm) Radicinin (sunlight)	0.5	24.510	2.618 ± 0.005	73.82
	0.5	24.513	0.110 ± 0.001	98.90
EPYR (standard)	1.0	17.381	-	-
EPYR (room temperature)	1.0	17.347	10.148 ± 0.029	49.26
EPYR (30 °C)	1.0	17.433	7.983 ± 0.014	60.09
EPYR (254 nm)	1.0	17.380	8.178 ± 0.014	59.11
EPYR (sunlight)	1.0	17.310	6.936 ± 0.012	65.32
Blank	1.0	-	-	-

## Data Availability

Not applicable.
